# Supplementary material for: Social exposome and brain health outcomes of dementia across Latin America
Source: Nat Commun. 2025 Sep 11;16:8196. doi: 10.1038/s41467-025-63277-6 (PMC12426228; doi:10.1038/s41467-025-63277-6)
Supplement: Supplementary file 1 — Supplementary Information [file 41467_2025_63277_MOESM1_ESM.pdf]

Social exposome and brain health outcomes of dementia across Latin America

Supplementary Information

Supplementary Information 1. Sensitivity analysis for country effects

We examined potential bias from country-specific effects to ensure that the observed associations between MSE and clinical and cognitive outcomes were not influenced by any country. The inclusion of multiple dummy variables[1-3] for factors such as country can be considered suboptimal, as it may introduce unnecessary complexity into SEM models[4]. Although such models may be well-specified and theoretically justified, they can still yield maladjusted fit indices[4]. Consequently, we conducted multiple linear regression analyses using MSE scores as predictors of cognition, functional ability, and neuropsychiatric symptoms, with country included as dummy covariates[5]. All models were statistically significant ($p < 0.001$) and MSE effects were replicated for cognition ($\beta = 0.91$, $p < 0.001$), functional ability ($\beta = 0.41$, $p < 0.001$), and neuropsychiatric symptoms ($\beta = 0.43$, $p < 0.001$).

Supplementary Information 2. Exclusion of education from the MSE global score

To eliminate potential bias in the association between MSE and cognition caused by education in HC, we created a model excluding this score. Results showed that the association between adverse MSE and reduced cognition persisted after excluding education ($\beta = 0.304$ [95% CI = 0.241- 0.367], $p < 0.001$). Similar indices were obtained as in the model that included education: CFI = 0.895, TLI = 0.721, RMSEA = 0.124, and SRMR = 0.083.

Supplementary Information 3. Comparison of MSE and SES models

We created a latent model of objective SES using educational attainment, occupation, assets, ability to cover basic needs, disposable income, proportion of income allocated to mortgage payments, and financial resilience. For a subjective SES model, we used self-evaluations of household socioeconomic position within their community based on income, education, and occupational status[6]. These objective and subjective SES factors were then used to predict cognition, functional ability, and neuropsychiatric symptoms.

For the entire sample, lower objective SES was linked to lower cognitive function (β = 0.185, 95% CI [0.135–0.235], p < 0.001) but did not predict functional ability (β = -0.009, 95% CI [-0.060–0.043], p = 0.744) or neuropsychiatric symptoms (β = 0.040, 95% CI [-0.011–0.091], p = 0.126). Regarding subjective SES, the latent factor was associated with decreased cognitive function (β = 0.093, 95% CI [0.048–0.138], p < 0.001), reduced functional ability (β = 0.133, 95% CI [0.088–0.177], p < 0.001), and increased neuropsychiatric symptoms (β = 0.097, 95% CI [0.052–0.142], p < 0.001).

Relative fit indices showed that the MSE model had a better fit[7] than the SES models, with AIC = -41641.99, BIC = -41379.74, and SABIC = -41525.88. The objective SES model had AIC = -20051.62, BIC = -19920.49, and SABIC = -19993.57. The subjective SES model showed SRMR = 0.026, AIC = -10629.72, BIC = -10544.20, and SABIC = -10591.86.

Supplementary Information 4. Functional connectivity analysis with AAL atlas

We conducted regression analyses via parametric tests with the MSE score as a predictor of the whole-brain ROI-to-ROI connectivity employing the AAL atlas. Like the main functional connectivity

analysis described in the manuscript, age, sex, and scanner effects were included as covariates of no interest. Each patient group was analyzed concurrently with healthy controls (HCs). All results were P_{FDR} corrected at the connection level, accounting for the size of the ROI-to-ROI matrix for each seed ROI, as implemented in the CONN toolbox[8].

Results replicated the main functional connectivity analysis, showing fronto-temporo-cerebellar associations and greater compensatory hyperconnectivity in AD than FTLD (Supplementary Tables 7-8). Cerebellar associations were more pronounced across groups, probably because the AAL atlas natively includes cerebellar regions[9], unlike the Brainnetome[10], which required an external addition of cerebellum.

Supplementary Information 5. Items removed from the MSE questionnaire to avoid circular associations with dementia

Certain items and options were excluded from the item grouping for MSE dimensions (see Scoring section) to prevent circular associations with dementia brain health outcomes, which may inflate the associations between these profiles and MSE. From the item "Now, please think about the homes you have lived in during different periods of your life. On the left side of the table below you will find a list of amenities that your different homes may or may not have had. Along the top of the table, we list the different life periods. For each life period, tell us if your home had the listed amenities (yes / no)" we removed the option "Smartphone". We did not consider the item "Are you currently working? Please consider paid and/or unpaid work". Lastly, for the item "What are your current sources of income? Please select all that apply" we discarded the option "Salary from my current job/employment".

Supplementary Tables

Indicator	β	*P* value	Lower 95% CI	Upper 95% CI
All subjects model				
Education	0.732	1×10^{-308}	0.685	0.778
Food insecurity	0.702	1×10^{-308}	0.658	0.745
Financial status	0.694	1×10^{-308}	0.659	0.73
Assets	0.649	1×10^{-308}	0.61	0.687
Access to healthcare	0.506	1×10^{-308}	0.456	0.557
Childhood labor	0.465	1×10^{-308}	0.426	0.504
Subjective SES	0.461	1×10^{-308}	0.422	0.499
Childhood experiences	0.443	1×10^{-308}	0.403	0.482
Traumatic events	0.221	1×10^{-15}	0.173	0.268
Relations	0.136	1×10^{-8}	0.089	0.182
HC model				
Education	0.921	1×10^{-8}	0.865	0.976
Food insecurity	0.373	1×10^{-9}	0.302	0.444
Financial status	0.526	1×10^{-9}	0.477	0.574
Assets	0.924	1×10^{-8}	0.88	0.967
Access to healthcare	0.219	1×10^{-5}	0.143	0.295
Childhood labor	0.44	1×10^{-7}	0.388	0.491
Subjective SES	0.191	1×10^{-5}	0.135	0.247
Childhood experiences	0.216	1×10^{-5}	0.157	0.275
Traumatic events	-0.023	0.529	-0.096	0.049

Relations	-0.002	0.942	-0.063	0.059
AD model				
Education	0.8	1×10^{-308}	0.73	0.869
Food insecurity	0.716	1×10^{-308}	0.65	0.782
Financial status	0.643	1×10^{-308}	0.583	0.703
Assets	0.683	1×10^{-308}	0.621	0.745
Access to healthcare	0.47	1×10^{-308}	0.388	0.552
Childhood labor	0.459	1×10^{-15}	0.393	0.524
Subjective SES	0.534	1×10^{-308}	0.476	0.593
Childhood experiences	0.484	1×10^{-16}	0.422	0.546
Traumatic events	0.283	1×10^{-8}	0.204	0.361
Relations	0.163	1×10^{-4}	0.085	0.241
FTLD model				
Education	0.796	1×10^{-13}	0.698	0.895
Food insecurity	0.62	1×10^{-308}	0.506	0.733
Financial status	0.691	1×10^{-308}	0.598	0.784
Assets	0.666	1×10^{-11}	0.569	0.763
Access to healthcare	0.626	1×10^{-308}	0.511	0.742
Childhood labor	0.501	1×10^{-9}	0.393	0.61
Subjective SES	0.675	1×10^{-12}	0.589	0.762
Childhood experiences	0.458	1×10^{-8}	0.348	0.567
Traumatic events	0.228	0.001	0.092	0.364
Relations	0.138	0.045	0.003	0.274

Supplementary Table 1. Indicators and statistics for each SEM model. No adjustments were made for multiple comparisons. The SEM were implemented as two-sided models by default.

Predictor	Coefficient			
	All	HC	AD	FTLD
Cognition				
Education	0.23	0.14	0.18	0.04
Food insecurity	.	-0.01	0.03	.
Financial status	-0.02	.	.	.
Assets	0.09	0.26	.	0.01
Access to healthcare	.	-0.06	0.02	.
Childhood labor	.	0.03	.	0.03
Subjective SES	0.01	-0.06	.	.
Childhood experiences	0.02	-0.04	0.01	0.08
Traumatic events	-0.43	-0.03	-0.10	.
Relations	.	-0.04	.	.
Functional ability				
Education	0.12	-	.	.
Food insecurity	0.02	-	.	.
Financial status	-0.10	-	.	0.17
Assets	-0.09	-	.	.
Access to healthcare	0.11	-	.	.
Childhood labor	-0.05	-	.	0.08
Subjective SES	0.16	-	0.08	.

Childhood experiences	0.05	-	.	0.05
Traumatic events	-0.70	-	.	.
Relations	0.11	-	.	0.15
Neuropsychiatric symptoms				
Education	.	-	.	.
Food insecurity	.	-	0.05	.
Financial status	.	-	.	0.13
Assets	-0.05	-	.	.
Access to healthcare	0.14	-	0.01	.
Childhood labor	-0.01	-	.	0.07
Subjective SES	0.05	-	.	.
Childhood experiences	0.09	-	0.05	.
Traumatic events	-0.09	-	.	0.29
Relations	0.04	-	.	0.03

Supplementary Table 2. Lasso regression coefficients. Dots indicate variables discarded for model performance. Hyphens indicate models not run due to non-significant associations with the outcome variable in the SEM model.

Regions	T value	P_{FDR}
Left superior frontal gyrus medial area 8 - left cingulate gyrus caudodorsal area 24	-4.79	0.001
Left superior frontal gyrus dorsolateral area 6 - left cingulate gyrus caudodorsal area 24	-3.49	0.021

Left superior frontal gyrus dorsolateral area 6 - left cingulate gyrus caudal area 23	-4.33	0.005
Left superior frontal gyrus medial area 8 - left cingulate gyrus caudal area 23	-3.25	0.048
Right middle frontal gyrus dorsal area 9/46 - left cingulate gyrus rostroventral area 24	-3.89	0.016
Left middle frontal gyrus dorsal area 9/46 - left cingulate gyrus rostroventral area 24	-3.61	0.032
Right occipital gyrus middle occipital gyrus - right middle temporal gyrus dorsolateral area37	-4.54	0.002
Left cuneus caudal cuneus gyrus - right middle temporal gyrus dorsolateral area37	-4.16	0.006
Right cuneus caudal lingual gyrus - right middle temporal gyrus dorsolateral area37	-4.1	0.014
Left superior frontal gyrus medial area 8 - right cingulate gyrus caudodorsal area 24	-3.94	0.009
Left middle frontal gyrus dorsal area 9/46 - right cingulate gyrus caudodorsal area 24	-3.52	0.032
Left superior frontal gyrus dorsolateral area 6 - right paracentral lobule area1/2/3 (lower limb region)	-3.55	0.019
Right superior frontal gyrus dorsolateral area 6 - right paracentral lobule area1/2/3 (lower limb region)	-3.76	0.023
Left superior frontal gyrus medial area 8 - right paracentral lobule area1/2/3 (lower limb region)	-3.21	0.049

Left superior frontal gyrus medial area 8 - right cingulate gyrus caudal area 23	-3.81	0.009
Right cingulate gyrus caudodorsal area 24 - left middle frontal gyrus dorsal area 9/46	-3.52	0.019
Left cingulate gyrus rostroventral area 24 - left middle frontal gyrus dorsal area 9/46	-3.6	0.024
Left cingulate gyrus caudodorsal area 24 - left middle frontal gyrus dorsal area 9/46	-3.23	0.027
Left superior frontal gyrus medial area 8 - right superior frontal gyrus dorsolateral area 6	-3.86	0.009
Left cingulate gyrus caudodorsal area 24 - right superior frontal gyrus dorsolateral area 6	-3.33	0.024
Left cingulate gyrus rostroventral area 24 - right middle frontal gyrus dorsal area 9/46	-3.89	0.016
Left thalamus occipital thalamus - left thalamus caudal temporal thalamus	-3.37	0.044
Right middle temporal gyrus dorsolateral area37 - right cuneus caudal lingual gyrus	-4.1	0.003
Right middle temporal gyrus dorsolateral area37 - right occipital gyrus middle occipital gyrus	-4.54	0.002
Left cuneus caudal cuneus gyrus - right occipital gyrus middle occipital gyrus	-3.95	0.008
Left cingulate gyrus caudal area 23 - left superior frontal gyrus dorsolateral area 6	-4.33	0.003

Left cingulate gyrus caudodorsal area 24 - left superior frontal gyrus dorsolateral area 6	-3.49	0.016
Left precuneus medial area 5(pem) - left superior frontal gyrus dorsolateral area 6	-3.73	0.038
Right middle temporal gyrus dorsolateral area37 - left superior frontal gyrus dorsolateral area 6	-2.85	0.048
Right middle temporal gyrus dorsolateral area37 - left cuneus caudal cuneus gyrus	-4.16	0.003
Right occipital gyrus middle occipital gyrus - left cuneus caudal cuneus gyrus	-3.95	0.008
Left cingulate gyrus caudal area 23 - left striatum dorsal caudate	-2.93	0.049
Left cingulate gyrus caudal area 23 - right precuneus medial area 5(pem)	-3.27	0.025
Left superior frontal gyrus medial area 8 - left precuneus medial area 5(pem)	-3.66	0.013
Left superior frontal gyrus dorsolateral area 6 - left precuneus medial area 5(pem)	-3.73	0.019
Right cingulate gyrus caudodorsal area 24 - right superior frontal gyrus medial area 8	-3.23	0.040
Left cingulate gyrus caudodorsal area 24 - right superior frontal gyrus medial area 8	-2.93	0.049
Left cingulate gyrus caudodorsal area 24 - left superior frontal gyrus medial area 8	-4.79	0.001
Right cingulate gyrus caudodorsal area 24 - left superior frontal gyrus medial area 8	-3.94	0.005

Right superior frontal gyrus dorsolateral area 6 - left superior frontal gyrus medial area 8	-3.86	0.023
Left cingulate gyrus caudal area 23 - left superior frontal gyrus medial area 8	-3.25	0.025
Left precuneus medial area 5(pem) - left superior frontal gyrus medial area 8	-3.66	0.038
Right cingulate gyrus caudal area 23 - left superior frontal gyrus medial area 8	-3.81	0.044
Left cingulate gyrus caudal area 23 - left superior parietal lobule caudal area 7	-5.44	0.000
Right middle temporal gyrus dorsolateral area37 - left inferior temporal gyrus extreme lateroventral area37	-4.18	0.003
Right middle temporal gyrus dorsolateral area37 - left fusiform gyrus medioventral area37	-4.09	0.003
Right middle temporal gyrus dorsolateral area37 - right superior occipital gyrus lateral superior occipital gyrus	-4.06	0.003
Right middle temporal gyrus dorsolateral area37 - left fusiform gyrus lateroventral area37	-4.01	0.003
Right cingulate gyrus caudodorsal area 24 - left precentral gyrus caudal ventrolateral area 6	-4.43	0.003
Right cingulate gyrus caudodorsal area 24 - right inferior frontal gyrus inferior frontal sulcus	-4.26	0.003
Left thalamus occipital thalamus - left parahippocampal gyrus area th (medial pphc)	-4.42	0.003

Right cingulate gyrus caudodorsal area 24 - right hippocampus caudal hipp	4.16	0.004
Left cingulate gyrus caudodorsal area 24 - left cerebellum 3	4.15	0.004
Left cingulate gyrus caudodorsal area 24 - left precentral gyrus caudal ventrolateral area 6	-4.1	0.004
Right middle frontal gyrus dorsal area 9/46 - left inferior frontal gyrus inferior frontal sulcus	-4.33	0.005
Left inferior frontal gyrus rostral area 45 - right inferior frontal gyrus rostral area 45	-4.32	0.005
Right cingulate gyrus caudodorsal area 24 - right middle frontal gyrus ventral area 9/46	-3.99	0.005
Right middle temporal gyrus dorsolateral area37 - right cuneus caudal cuneus gyrus	-3.78	0.006
Right middle temporal gyrus dorsolateral area37 - left superior occipital gyrus lateral superior occipital gyrus	-3.74	0.006
Right middle temporal gyrus dorsolateral area37 - left cuneus caudal lingual gyrus	-3.72	0.006
Left cuneus caudal cuneus gyrus - left inferior temporal gyrus ventrolateral area 37	-4.11	0.006
Right middle temporal gyrus dorsolateral area37 - right superior occipital gyrus medial superior occipital gyrus	-3.68	0.007
Right middle temporal gyrus dorsolateral area37 - left occipital gyrus middle occipital gyrus	-3.64	0.007
Right occipital gyrus middle occipital gyrus - left superior temporal gyrus caudal area 22	-4.06	0.008

Left superior frontal gyrus medial area 8 - left precentral gyrus caudal dorsolateral area 6	-3.84	0.009
Right middle temporal gyrus dorsolateral area37 - right occipital gyrus inferior occipital gyrus	-3.55	0.009
Left cingulate gyrus caudal area 23 - right hippocampus caudal hipp	3.92	0.009
Right cingulate gyrus caudodorsal area 24 - left cerebellum 3	3.71	0.011
Left cingulate gyrus caudal area 23 - left angular gyrus caudal area 39(pgp)	-3.81	0.011
Right middle temporal gyrus dorsolateral area37 - left cuneus rostral lingual gyrus	-3.47	0.011
Left striatum dorsal caudate - left inferior frontal gyrus ventral area 44	-4.14	0.011
Left cuneus caudal cuneus gyrus - right inferior temporal gyrus ventrolateral area 37	-3.79	0.012
Right middle temporal gyrus dorsolateral area37 - right cuneus rostral lingual gyrus	-3.39	0.014
Right middle temporal gyrus dorsolateral area37 - left cuneus rostral cuneus gyrus	-3.37	0.014
Right middle temporal gyrus dorsolateral area37 - right inferior temporal gyrus ventrolateral area 37	-3.35	0.014
Left superior frontal gyrus dorsolateral area 6 - left paracentral lobule area1/2/3 (lower limb region)	-3.92	0.014
Left cingulate gyrus caudal area 23 - left supramarginal gyrus rostrodorsal area 40(pft)	-3.67	0.015
Right middle temporal gyrus dorsolateral area37 - right cuneus rostral cuneus gyrus	-3.31	0.015

Left cingulate gyrus caudal area 23 - left precuneus medial area 7(pep)	-3.59	0.016
Left cingulate gyrus caudal area 23 - left superior parietal lobule rostral area 7	-3.56	0.016
Left cingulate gyrus rostroventral area 24 - right middle frontal gyrus ventral area 9/46	-3.94	0.016
Left thalamus occipital thalamus - left parahippocampal gyrus caudal area 35/36	-3.89	0.016
Left cingulate gyrus caudodorsal area 24 - right hippocampus caudal hipp	3.69	0.016
Left cingulate gyrus caudodorsal area 24 - right precentral gyrus caudal dorsolateral area 6	-3.55	0.016
Left cingulate gyrus caudodorsal area 24 - right cerebellum 3	3.55	0.016
Left cingulate gyrus caudodorsal area 24 - left middle frontal gyrus ventrolateral area 6	-3.53	0.016
Left cingulate gyrus caudodorsal area 24 - left inferior frontal gyrus dorsal area 44	-3.51	0.016
Right middle temporal gyrus dorsolateral area37 - right fusiform gyrus medioventral area37	-3.27	0.017
Left inferior frontal gyrus rostral area 45 - left striatum dorsolateral putamen	-3.87	0.017
Right middle temporal gyrus dorsolateral area37 - right occipital gyrus occipital polar cortex	-3.24	0.017
Right middle temporal gyrus dorsolateral area37 - left occipital gyrus area v5/mt	-3.23	0.017

Left superior frontal gyrus dorsolateral area 6 - left supramarginal gyrus rostrodorsal area 40(pft)	-3.64	0.019
Left superior frontal gyrus dorsolateral area 6 - left paracentral lobule area 4, (lower limb region)	-3.61	0.019
Left cingulate gyrus rostroventral area 24 - left parahippocampal gyrus area ti(temporal agranular insular cortex)	3.72	0.020
Left thalamus occipital thalamus - left cingulate gyrus ventral area 23	-3.71	0.021
Left cingulate gyrus caudal area 23 - right superior parietal lobule caudal area 7	-3.43	0.021
Left cingulate gyrus caudal area 23 - left middle frontal gyrus ventrolateral area 6	-3.41	0.021
Right superior frontal gyrus dorsolateral area 6 - left paracentral lobule area1/2/3 (lower limb region)	-3.69	0.023
Right cingulate gyrus caudodorsal area 24 - right middle frontal gyrus ventrolateral area 6	-3.42	0.024
Left cingulate gyrus caudodorsal area 24 - left hippocampus caudal hipp	3.32	0.024
Left cingulate gyrus caudodorsal area 24 - right superior frontal gyrus medial area 6	-3.3	0.024
Left cingulate gyrus caudal area 23 - left superior parietal lobule lateral area 5	-3.35	0.024
Right middle temporal gyrus dorsolateral area37 - vermis 6	-3.11	0.025
Left cingulate gyrus caudal area 23 - right superior parietal lobule intraparietal area 7(hip3)	-3.25	0.025
Left cingulate gyrus caudal area 23 - right precuneus medial area 7(pep)	-3.24	0.025

Left striatum dorsal caudate - right cerebellum 3	3.76	0.027
Left inferior frontal gyrus rostral area 45 - right precentral gyrus area 4(tongue and larynx region)	-3.64	0.028
Right middle temporal gyrus dorsolateral area37 - right supramarginal gyrus rostroventral area 40(pfop)	-3.02	0.031
Left middle frontal gyrus dorsal area 9/46 - right superior frontal gyrus medial area 6	-3.86	0.032
Left middle frontal gyrus dorsal area 9/46 - right superior parietal lobule postcentral area 7	-3.67	0.032
Left cingulate gyrus caudal area 23 - right superior parietal lobule rostral area 7	-3.14	0.032
Left cingulate gyrus caudal area 23 - right superior parietal lobule postcentral area 7	-3.11	0.032
Left cingulate gyrus caudal area 23 - right superior occipital gyrus lateral superior occipital gyrus	-3.11	0.032
Right middle temporal gyrus dorsolateral area37 - right occipital gyrus area v5/mt	-2.98	0.035
Left cuneus caudal cuneus gyrus - right fusiform gyrus medioventral area37	-3.43	0.036
Left cingulate gyrus caudodorsal area 24 - left middle frontal gyrus inferior frontal junction	-3.1	0.037
Left cingulate gyrus caudodorsal area 24 - left paracentral lobule area1/2/3 (lower limb region)	-3.1	0.037
Left cingulate gyrus caudodorsal area 24 - left inferior frontal gyrus inferior frontal sulcus	-3.07	0.037

Left cingulate gyrus caudodorsal area 24 - right middle frontal gyrus ventrolateral area 6	-3.06	0.037
Left cingulate gyrus caudodorsal area 24 - left middle frontal gyrus ventral area 9/46	-3.05	0.037
Left cingulate gyrus caudal area 23 - right precentral gyrus caudal dorsolateral area 6	-3.04	0.038
Right superior frontal gyrus dorsolateral area 6 - right inferior frontal gyrus inferior frontal sulcus	-3.45	0.042
Right occipital gyrus middle occipital gyrus - left striatum nucleus accumbens	-3.45	0.042
Right middle temporal gyrus dorsolateral area37 - left occipital gyrus inferior occipital gyrus	-2.9	0.043
Left thalamus occipital thalamus - left precuneus dorsomeidal parietooccipital sulcus(per)	-3.41	0.044
Right cingulate gyrus caudodorsal area 24 - left middle frontal gyrus ventrolateral area 6	-3.17	0.045
Right middle frontal gyrus dorsal area 9/46 - left cingulate gyrus pregenual area 32	-3.51	0.045
Left cingulate gyrus caudodorsal area 24 - left caudodorsal posterior insula hypergranular insula	2.92	0.049
Left cingulate gyrus caudodorsal area 24 - right superior occipital gyrus medial superior occipital gyrus	-2.9	0.049
Left cingulate gyrus caudodorsal area 24 - vermis 3	2.9	0.049

Right cingulate gyrus caudodorsal area 24 - right inferior frontal gyrus dorsal area 44	-3.11	0.049
Left superior frontal gyrus dorsolateral area 6 - left precentral gyrus caudal ventrolateral area 6	-3.17	0.049
Left superior frontal gyrus dorsolateral area 6 - left postcentral gyrus area 2	-3.17	0.049
Left cingulate gyrus caudal area 23 - left superior occipital gyrus lateral superior occipital gyrus	-2.93	0.049

Supplementary Table 3. MSE on ROI-to-ROI functional connectivity for AD. $P_{FDR}<0.05$, covariates: age, sex, and recording site.

Regions	T value	P_{FDR}
Right cingulate gyrus pregenual area 32 - right hippocampus caudal hipp	4.49	0.002
Left inferior frontal gyrus inferior frontal sulcus - right hippocampus caudal hipp	4.48	0.003
Left inferior frontal gyrus dorsal area 44 - right hippocampus caudal hipp	3.84	0.021
Right hippocampus caudal hipp - right cingulate gyrus pregenual area 32	4.49	0.002
Left hippocampus caudal hipp - right cingulate gyrus pregenual area 32	4.27	0.002
Left superior temporal gyrus medial area 38 - right cingulate gyrus pregenual area 32	3.78	0.007
Left inferior frontal gyrus inferior frontal sulcus - right cingulate gyrus pregenual area 32	-3.36	0.025
Left parahippocampal gyrus area 28/34 (ec, entorhinal cortex) - right cingulate gyrus pregenual area 32	3.38	0.045

Right parahippocampal gyrus area th (medial pphc) - right cingulate gyrus pregenual area 32	3.43	0.048
Right cingulate gyrus pregenual area 32 - left hippocampus caudal hipp	4.27	0.002
Left superior temporal gyrus medial area 38 - left hippocampus caudal hipp	-4.45	0.002
Left cingulate gyrus pregenual area 32 - left hippocampus caudal hipp	4.48	0.003
Left middle frontal gyrus inferior frontal junction - left hippocampus caudal hipp	3.78	0.026
Left inferior frontal gyrus inferior frontal sulcus - left hippocampus caudal hipp	3.16	0.033
Right superior frontal gyrus medial area 9 - left hippocampus caudal hipp	3.68	0.034
Right hippocampus caudal hipp - left inferior frontal gyrus inferior frontal sulcus	4.48	0.002
Right parahippocampal gyrus area th (medial pphc) - left inferior frontal gyrus inferior frontal sulcus	3.72	0.022
Right cingulate gyrus pregenual area 32 - left inferior frontal gyrus inferior frontal sulcus	-3.36	0.024
Left hippocampus caudal hipp - left inferior frontal gyrus inferior frontal sulcus	3.16	0.026
Left hippocampus caudal hipp - left cingulate gyrus pregenual area 32	4.48	0.002
Right hippocampus caudal hipp - left cingulate gyrus pregenual area 32	3.2	0.033
Right cingulate gyrus pregenual area 32 - left parahippocampal gyrus area 28/34 (ec, entorhinal cortex)	3.38	0.024
Left hippocampus caudal hipp - left middle frontal gyrus inferior frontal junction	3.78	0.009
Right hippocampus caudal hipp - left inferior frontal gyrus dorsal area 44	3.84	0.010
Left hippocampus caudal hipp - left inferior frontal gyrus dorsal area 44	3.34	0.022

Right cingulate gyrus pregenual area 32 - left inferior frontal gyrus dorsal area 44	-3.3	0.027
Left superior temporal gyrus medial area 38 - right superior frontal gyrus medial area 9	3.79	0.007
Left hippocampus caudal hipp - right superior frontal gyrus medial area 9	3.68	0.011
Right hippocampus caudal hipp - right superior frontal gyrus medial area 9	3.16	0.033
Right hippocampus caudal hipp - left striatum dorsal caudate	3.29	0.033
Left hippocampus caudal hipp - left superior temporal gyrus medial area 38	-4.45	0.002
Right cingulate gyrus pregenual area 32 - left superior temporal gyrus medial area 38	3.78	0.010
Right superior frontal gyrus medial area 9 - left superior temporal gyrus medial area 38	3.79	0.034
Left inferior frontal gyrus inferior frontal sulcus - right parahippocampal gyrus area th (medial pphc)	3.72	0.013
Right cingulate gyrus pregenual area 32 - right parahippocampal gyrus area th (medial pphc)	3.43	0.024
Left cingulate gyrus pregenual area 32 - left parahippocampal gyrus rostral area 35/36	3.47	0.027
Right cingulate gyrus pregenual area 32 - left parahippocampal gyrus rostral area 35/36	3.06	0.047
Right cingulate gyrus pregenual area 32 - left cingulate gyrus ventral area 23	4.35	0.002
Right cingulate gyrus pregenual area 32 - left parahippocampal gyrus area th (medial pphc)	4.27	0.002
Left superior temporal gyrus medial area 38 - left thalamus sensory thalamus	-4.43	0.002

Right hippocampus caudal hipp - left cerebellum 6	-4.29	0.002
Left middle frontal gyrus inferior frontal junction - left superior frontal gyrus medial area 6	-4.46	0.003
Left superior temporal gyrus medial area 38 - right precentral gyrus caudal ventrolateral area 6	4.11	0.005
Left hippocampus caudal hipp - right inferior frontal gyrus dorsal area 44	3.96	0.006
Left hippocampus caudal hipp - left superior frontal gyrus medial area 10	3.93	0.006
Left superior temporal gyrus medial area 38 - right middle frontal gyrus inferior frontal junction	3.98	0.006
Left superior temporal gyrus medial area 38 - left cingulate gyrus caudodorsal area 24	3.92	0.006
Left parahippocampal gyrus area 28/34 (ec, entorhinal cortex) - left cerebellum 4 5	-4.3	0.007
Left inferior frontal gyrus inferior frontal sulcus - right cerebellum 4 5	4.02	0.008
Left inferior frontal gyrus inferior frontal sulcus - right cerebellum 3	3.99	0.008
Right hippocampus caudal hipp - right paracentral lobule area1/2/3 (lower limb region)	3.8	0.010
Right hippocampus caudal hipp - right middle frontal gyrus dorsal area 9/46	3.74	0.010
Right cingulate gyrus pregenual area 32 - left superior temporal gyrus lateral area 38	3.69	0.012
Left superior temporal gyrus medial area 38 - right cingulate gyrus caudodorsal area 24	3.59	0.013
Left inferior frontal gyrus inferior frontal sulcus - right parahippocampal gyrus area tl (lateral pphc, posterior parahippocampal gyrus)	3.72	0.013

Left hippocampus caudal hipp - right middle frontal gyrus inferior frontal junction	3.59	0.013
Left inferior frontal gyrus inferior frontal sulcus - right hippocampus rostral hipp	3.65	0.013
Left inferior frontal gyrus inferior frontal sulcus - left thalamus sensory thalamus	3.62	0.013
Left hippocampus caudal hipp - left inferior temporal gyrus rostral area 20	-3.55	0.014
Right parahippocampal gyrus area th (medial pphc) - right cingulate gyrus caudodorsal area 24	4.07	0.016
Right parahippocampal gyrus area th (medial pphc) - right precentral gyrus caudal ventrolateral area 6	3.9	0.016
Left striatum dorsal caudate - left cerebellum 3	4.07	0.017
Left hippocampus caudal hipp - left cerebellum 6	-3.43	0.017
Left hippocampus caudal hipp - left cingulate gyrus rostroventral area 24	3.43	0.017
Right hippocampus caudal hipp - right inferior frontal gyrus dorsal area 44	3.54	0.018
Left cingulate gyrus pregenual area 32 - left parahippocampal gyrus area th (medial pphc)	3.73	0.021
Left cingulate gyrus pregenual area 32 - right parahippocampal gyrus area tl (lateral pphc, posterior parahippocampal gyrus)	3.7	0.021
Left cingulate gyrus pregenual area 32 - left precuneus dorsomeidal parietooccipital sulcus(per)	3.63	0.021
Left cingulate gyrus pregenual area 32 - left parahippocampal gyrus area tl (lateral pphc, posterior parahippocampal gyrus)	3.6	0.021
Left inferior frontal gyrus dorsal area 44 - right amygdala lateral amyg	3.92	0.021

Left superior temporal gyrus medial area 38 - left thalamus posterior parietal thalamus	-3.43	0.021
Left inferior frontal gyrus inferior frontal sulcus - left fusiform gyrus rostroventral area 20	3.45	0.022
Left inferior frontal gyrus dorsal area 44 - right hippocampus rostral hipp	3.71	0.023
Left hippocampus caudal hipp - left middle frontal gyrus ventral area 9/46	3.3	0.023
Left hippocampus caudal hipp - right middle frontal gyrus dorsal area 9/46	3.26	0.024
Left hippocampus caudal hipp - right cingulate gyrus caudal area 23	3.24	0.024
Right cingulate gyrus pregenual area 32 - right superior temporal gyrus medial area 38	3.37	0.024
Left inferior frontal gyrus inferior frontal sulcus - right cerebellum 9	3.35	0.025
Left inferior frontal gyrus inferior frontal sulcus - left parahippocampal gyrus area th (medial pphc)	3.33	0.025
Left hippocampus caudal hipp - right parahippocampal gyrus caudal area 35/36	-3.2	0.026
Left hippocampus caudal hipp - right precentral gyrus caudal ventrolateral area 6	3.15	0.026
Left hippocampus caudal hipp - left cerebeul crus 1	-3.15	0.026
Left middle frontal gyrus inferior frontal junction - right superior frontal gyrus medial area 6	-3.66	0.027
Right superior temporal gyrus te1.0 and te1.2 - right middle temporal gyrus caudal area 21	-3.94	0.028
Right hippocampus caudal hipp - right middle frontal gyrus ventrolateral area 6	3.38	0.028
Left hippocampus caudal hipp - left superior frontal gyrus medial area 9	3.11	0.029

Left parahippocampal gyrus area 28/34 (ec, entorhinal cortex) - right recerebul 4 5	-3.75	0.029
Left hippocampus caudal hipp - left middle temporal gyrus rostral area 21	-3.07	0.031
Left hippocampus caudal hipp - left fusiform gyrus rostroventral area 20	-3.04	0.033
Left inferior frontal gyrus inferior frontal sulcus - left superior frontal gyrus medial area 6	-3.2	0.033
Left inferior frontal gyrus inferior frontal sulcus - left middle frontal gyrus dorsal area 9/46	-3.17	0.033
Left inferior frontal gyrus inferior frontal sulcus - left inferior temporal gyrus intermediate ventral area 20	3.15	0.033
Right hippocampus caudal hipp - left superior frontal gyrus medial area 10	3.26	0.033
Right hippocampus caudal hipp - right cerebellum 6	-3.24	0.033
Right hippocampus caudal hipp - right superior temporal gyrus area 41/42	-3.15	0.033
Right hippocampus caudal hipp - right thalamus posterior parietal thalamus	-3.15	0.033
Right hippocampus caudal hipp - vermis 6	-3.13	0.033
Right superior frontal gyrus medial area 9 - right thalamus posterior parietal thalamus	3.53	0.034
Right superior frontal gyrus medial area 9 - left superior frontal gyrus dorsolateral area 6	-3.48	0.034
Right superior frontal gyrus medial area 9 - right superior temporal gyrus medial area 38	3.46	0.034
Right superior temporal gyrus te1.0 and te1.2 - right orbital gyrus medial area 11	-3.67	0.035

Right superior temporal gyrus TE1.0 and TE1.2 - right superior frontal gyrus medial area 6	3.59	0.035
Left inferior frontal gyrus inferior frontal sulcus - right fusiform gyrus medioventral area37	3.11	0.035
Right hippocampus caudal hipp - right thalamus occipital thalamus	-3.09	0.035
Left hippocampus caudal hipp - right superior frontal gyrus medial area 10	3	0.035
Right cingulate gyrus pregenual area 32 - left amygdala medial amyg	3.19	0.036
Right cingulate gyrus pregenual area 32 - right hippocampus rostral hipp	3.17	0.036
Left superior temporal gyrus medial area 38 - left superior frontal gyrus medial area 9	3.22	0.039
Left hippocampus caudal hipp - left precentral gyrus caudal ventrolateral area 6	2.95	0.039
Left hippocampus caudal hipp - right amygdala lateral amyg	-2.92	0.042
Left hippocampus caudal hipp - left inferior temporal gyrus caudolateral of area 20	-2.9	0.042
Left inferior frontal gyrus inferior frontal sulcus - right fusiform gyrus lateroventral area37	3.02	0.043
Left inferior frontal gyrus inferior frontal sulcus - right superior frontal gyrus medial area 8	-3.01	0.043
Left inferior frontal gyrus inferior frontal sulcus - left cingulate gyrus caudal area 23	-2.98	0.045
Left parahippocampal gyrus area 28/34 (ec, entorhinal cortex) - vermis 3	-3.47	0.045
Left parahippocampal gyrus area 28/34 (ec, entorhinal cortex) - left thalamus caudal temporal thalamus	-3.38	0.045
Left hippocampus caudal hipp - left cingulate gyrus caudal area 23	2.86	0.046

Left inferior frontal gyrus inferior frontal sulcus - left cerebellum 4 5	2.94	0.048
Right hippocampus caudal hipp - left thalamus occipital thalamus	-2.97	0.048
Right superior frontal gyrus medial area 9 - left parahippocampal gyrus area th (medial pphc)	3.28	0.048
Right superior frontal gyrus medial area 9 - right thalamus occipital thalamus	3.23	0.048
Right superior frontal gyrus medial area 9 - right cerebellum 10	3.22	0.048
Left hippocampus caudal hipp - right cerebellum 10	-2.83	0.049

Supplementary Table 4. MSE on ROI-to-ROI functional connectivity for FTLD. $P_{FDR}<0.05$, covariates: age, sex, and recording site.

Association	β	*P* value	Lower 5% CI	Upper 95% CI
All subjects model (CFI = 0.930, TLI = 0.866, RMSEA = 0.076, SRMR = 0.051)				
Age → MSE	0.015	0.516	-0.031	0.061
Sex → MSE	0.032	0.169	-0.013	0.078
MSE → Cognition	0.254	1×10^{-308}	0.212	0.296
MSE → Functional ability	0.064	0.004	0.021	0.107
MSE → Neuropsychiatric symptoms	0.08	0.001	0.036	0.125
AD & FTLD model (CFI = 0.935, TLI = 0.888, RMSEA = 0.056, SRMR = 0.043)				
Age → MSE	0.036	0.598	-0.098	0.171
Sex → MSE	0.033	0.335	-0.034	0.099
Disease severity	-0.035	0.311	-0.101	0.032
Age at diagnosis	0.097	0.182	-0.044	0.237
Years after diagnosis	0.062	0.108	-0.013	0.137

MSE → Cognition	0.168	1×10^{-7}	0.212	0.296
MSE → Functional ability	0.08	0.004	0.021	0.107
MSE → Neuropsychiatric symptoms	0.132	1×10^{-4}	0.036	0.125
FTLD model (CFI = 0.987, TLI = 0.980, RMSEA = 0.029, SRMR = 0.043)				
FTLD subtype → MSE	0.029	0.68	-0.107	0.164
MSE → Cognition	0.171	0.014	0.041	0.301
MSE → Functional ability	0.175	0.013	0.044	0.307
MSE → Neuropsychiatric symptoms	0.16	0.019	0.032	0.288

Supplementary Table 5. Indicators and statistics for each sensitivity analysis-SEM model. No adjustments were made for multiple comparisons. The SEM were implemented as two-sided models by default.

Group	MRI	fMRI	
	Spatial signal-to-noise ratio	Spatial signal-to-noise ratio	Temporal signal-to-noise ratio
All Subjects	r = 0.056, p = 0.141	r = -0.063, p = 0.183	*r* = 0.005, p = 0.912
AD + CN	r = 0.056, p = 0.141	r = -0.082, p = 0.113	*r* = 0.017, p = 0.738
FTLD + CN	r = 0.015, p = 0.731	r = -0.026, p = 0.681	*r* = 0.005, p = 0.934

Supplementary Table 6. Pearson's correlations between MSE and MRI/fMRI data quality metrics. Correlation models were implemented as two-sided. No adjustments were made for multiple comparisons.

Regions	T value	P_{FDR}
Left striatum dorsal caudate - left cingulate gyrus caudodorsal area 24	-2.95	0.042
Left striatum dorsal caudate - left striatum dorsolateral putamen	-4.55	0.001
Left striatum dorsal caudate - left striatum globus pallidus	-4.54	0.001
Left striatum dorsal caudate - left inferior frontal gyrus ventral area 44	-4.27	0.002
Right cingulate gyrus caudodorsal area 24 - left precentral gyrus caudal ventrolateral area 6	-4.12	0.004
Right cingulate gyrus caudodorsal area 24 - right inferior frontal gyrus inferior frontal sulcus	-4.12	0.004
Right cingulate gyrus caudodorsal area 24 - left superior frontal gyrus medial area 8	-4.1	0.004
Right cingulate gyrus caudodorsal area 24 - right hippocampus caudal hipp	3.97	0.004
Right cingulate gyrus caudodorsal area 24 - left middle frontal gyrus dorsal area 9/46	-3.96	0.004
Right cingulate gyrus caudodorsal area 24 - right middle frontal gyrus ventral area 9/46	-3.95	0.004
Right cingulate gyrus caudodorsal area 24 - right inferior frontal gyrus ventral area 44	-3.95	0.004
Left striatum dorsal caudate - left striatum ventromedial putamen	-4.02	0.005
Left striatum dorsal caudate - right cerebellum 3	3.84	0.008
Left cingulate gyrus caudodorsal area 24 - left superior frontal gyrus medial area 8	-4.23	0.008
Left cingulate gyrus rostroventral area 24 - left middle frontal gyrus dorsal area 9/46	-4.16	0.010
Left cingulate gyrus rostroventral area 24 - right middle frontal gyrus dorsal area 9/46	-4.02	0.010

Left cingulate gyrus caudodorsal area 24 - left precentral gyrus caudal ventrolateral area 6	-3.85	0.012
Left cingulate gyrus caudodorsal area 24 - right hippocampus caudal hipp	3.85	0.012
Left striatum dorsal caudate - right striatum dorsolateral putamen	-3.62	0.013
Left striatum dorsal caudate - right striatum globus pallidus	-3.54	0.013
Left striatum dorsal caudate - left superior frontal gyrus medial area 8	-3.53	0.013
Left striatum dorsal caudate - left superior frontal gyrus medial area 9	-3.5	0.013
Left striatum dorsal caudate - left cingulate gyrus caudal area 23	-3.49	0.013
Left striatum dorsal caudate - left middle frontal gyrus inferior frontal junction	-3.49	0.013
Right cingulate gyrus caudodorsal area 24 - right striatum dorsal caudate	-3.52	0.016
Left striatum dorsal caudate - left middle frontal gyrus dorsal area 9/46	-3.38	0.018
Left striatum dorsal caudate - right fusiform gyrus rostroventral area 20	3.36	0.018
Left striatum dorsal caudate - right cingulate gyrus caudal area 23	-3.29	0.020
Left striatum dorsal caudate - right thalamus premotor thalamus	-3.29	0.020
Left striatum dorsal caudate - left parahippocampal gyrus area ti(temporal agranular insular cortex)	3.25	0.021
Left striatum dorsal caudate - left superior frontal gyrus dorsolateral area 8	-3.18	0.025
Right cingulate gyrus caudodorsal area 24 - left inferior frontal gyrus dorsal area 44	-3.34	0.025
Right cingulate gyrus caudodorsal area 24 - right superior frontal gyrus medial area 8	-3.34	0.025
Right cingulate gyrus caudodorsal area 24 - right parahippocampal gyrus area th (medial pphc)	3.29	0.026
Right cingulate gyrus caudodorsal area 24 - left hippocampus caudal hipp	3.28	0.026
Left cingulate gyrus caudodorsal area 24 - left hippocampus caudal hipp	3.58	0.026

Left striatum dorsal caudate - left precentral gyrus caudal ventrolateral area 6	-3.12	0.030
Left striatum dorsal caudate - right superior frontal gyrus medial area 9	-3.08	0.032
Left cingulate gyrus caudodorsal area 24 - left cerebellum 3	3.41	0.036
Left cingulate gyrus caudodorsal area 24 - left inferior frontal gyrus dorsal area 44	-3.39	0.036
Right cingulate gyrus caudodorsal area 24 - left middle frontal gyrus ventral area 9/46	-3.16	0.036
Left striatum dorsal caudate - left middle frontal gyrus ventrolateral area 8	-3.02	0.036
Right cingulate gyrus caudodorsal area 24 - right amygdala lateral amyg	3.13	0.037
Left striatum dorsal caudate - left middle frontal gyrus ventrolateral area 6	-2.98	0.039
Left cingulate gyrus caudodorsal area 24 - right superior frontal gyrus dorsolateral area 6	-3.25	0.045
Left cingulate gyrus caudodorsal area 24 - right inferior frontal gyrus ventral area 44	-3.24	0.045
Right cingulate gyrus caudodorsal area 24 - left superior frontal gyrus dorsolateral area 6	-3.03	0.045
Right cingulate gyrus caudodorsal area 24 - left cerebellum 3	3.02	0.045
Left striatum dorsal caudate - right parahippocampal gyrus rostral area 35/36	2.9	0.046
Left cingulate gyrus caudodorsal area 24 - left superior frontal gyrus dorsolateral area 6	-3.19	0.047
Left cingulate gyrus caudodorsal area 24 - right striatum dorsal caudate	-3.15	0.048

Supplementary Table 7. MSE on ROI-to-ROI functional connectivity for AD excluding subjects with less than 70% of artifact-free frames. P_{FDR}<0.05, covariates: age, sex, and recording site.

Regions	T value	P_{FDR}
Left hippocampus caudal hipp - right cingulate gyrus pregenual area 32	4.12	0.005

Right hippocampus caudal hipp - right cingulate gyrus pregenual area 32	3.96	0.013
Right cingulate gyrus pregenual area 32 - right hippocampus caudal hipp	3.96	0.007
Right cingulate gyrus pregenual area 32 - left hippocampus caudal hipp	4.12	0.005
Left hippocampus caudal hipp - left cingulate gyrus pregenual area 32	4.44	0.004
Left hippocampus caudal hipp - left superior temporal gyrus medial area 38	-4.28	0.004
Right cingulate gyrus pregenual area 32 - left parahippocampal gyrus area th (medial pphc)	4.43	0.004
Right cingulate gyrus pregenual area 32 - left cingulate gyrus ventral area 23	4.09	0.005
Left hippocampus caudal hipp - left superior frontal gyrus medial area 10	3.97	0.006
Right hippocampus caudal hipp - left cerebellum 6	-4.23	0.009
Left hippocampus caudal hipp - left middle frontal gyrus inferior frontal junction	3.76	0.012
Right hippocampus caudal hipp - left inferior frontal gyrus inferior frontal sulcus	3.83	0.015
Right cingulate gyrus pregenual area 32 - left parahippocampal gyrus area 28/34 (ec, entorhinal cortex)	3.69	0.015
Left hippocampus caudal hipp - right superior frontal gyrus medial area 9	3.57	0.019
Right hippocampus caudal hipp - right paracentral lobule area1/2/3 (lower limb region)	3.68	0.020
Left hippocampus caudal hipp - right inferior frontal gyrus dorsal area 44	3.52	0.020
Left hippocampus caudal hipp - right middle frontal gyrus inferior frontal junction	3.38	0.028
Left hippocampus caudal hipp - left cingulate gyrus rostroventral area 24	3.34	0.029
Left hippocampus caudal hipp - left inferior temporal gyrus rostral area 20	-3.27	0.032
Left hippocampus caudal hipp - left cerebellum 6	-3.25	0.032
Right cingulate gyrus pregenual area 32 - left parahippocampal gyrus caudal area 35/36	3.4	0.035

Right cingulate gyrus pregenual area 32 - left superior temporal gyrus medial area 38	3.34	0.037
Left hippocampus caudal hipp - left middle frontal gyrus ventral area 9/46	3.17	0.037
Left hippocampus caudal hipp - left cerebellum crus 1	-3.15	0.037
Left hippocampus caudal hipp - left superior frontal gyrus medial area 9	3.13	0.037
Left hippocampus caudal hipp - left inferior frontal gyrus dorsal area 44	3.1	0.038
Left hippocampus caudal hipp - left inferior frontal gyrus inferior frontal sulcus	3.08	0.038
Left hippocampus caudal hipp - right middle frontal gyrus dorsal area 9/46	3.03	0.042
Left hippocampus caudal hipp - left cingulate gyrus caudal area 23	3.02	0.042
Left hippocampus caudal hipp - left precentral gyrus caudal ventrolateral area 6	3.01	0.042
Right cingulate gyrus pregenual area 32 - left superior temporal gyrus lateral area 38	3.24	0.046
Right cingulate gyrus pregenual area 32 - left parahippocampal gyrus rostral area 35/36	3.2	0.047
Right cingulate gyrus pregenual area 32 - right parahippocampal gyrus area th (medial pphc)	3.16	0.048

Supplementary Table 8. MSE on ROI-to-ROI functional connectivity for FTLD excluding subjects with less than 70% of artifact-free frames. $P_{FDR}<0.05$, covariates: age, sex, and recording site.

Region	Coordinates			K_E	Peak P_{FDR}	TFCE
	X	Y	Z			
Frontal Inf Orb L	-48	36	-7.5	133756	0.000293566	10903.94141
Temporal Mid R	61.5	-4.5	-19.5	641	0.000293566	477.0377502
Precentral L	-43.5	-1.5	54	42	0.000293566	144.9977722
Frontal Mid R	34.5	19.5	54	39	0.000508227	114.6467056

Precuneus L	-7.5	-67.5	58.5	15	0.000293566	86.73767853
Frontal Mid R	39	16.5	55.5	45	0.000293566	85.63156128
Precuneus R	10.5	-67.5	63	48	0.000508227	80.21271515
Parietal Inf L	-48	-33	43.5	66	0.000945624	63.40354156
Parietal Inf L	-49.5	-27	45	30	0.001162407	56.42485046
Angular R	37.5	-69	48	24	0.000729234	54.45516968
Precuneus R	10.5	-54	72	27	0.000729234	50.7036171
Occipital Sup R	25.5	-78	45	20	0.002829853	25.75318527
Lingual R	12	-96	-7.5	17	0.016740975	19.79795647
Cuneus R	12	-82.5	40.5	25	0.022611542	18.5773983

Supplementary table 9. MSE on minimum-maximum-normalized voxel intensity values by scanner type for AD. P_{FDR}<0.05, TFCE-corrected, covariates: age, sex, TIV, and recording site. Regions are presented on MNI space using the AAL atlas.

Region	Coordinates			KE	Peak P_{FDR}	TFCE
	X	Y	Z			
Cerebellum 8 R	22.5	-72	-54	115063	0.00027952	8584.11816
Parietal Inf L	-39	-55.5	48	23	0.00049265	2683.32642
Frontal Sup Medial L	-6	42	52.5	397	0.00027952	740.144287
Frontal Sup L	-21	33	54	110	0.00027952	509.761871
Precentral L	-57	-4.5	33	205	0.00027952	368.110504
Precentral L	-46.5	7.5	49.5	343	0.00027952	307.056244
Precentral L	-43.5	-1.5	54	106	0.00027952	248.53682
Precuneus R	10.5	-54	72	98	0.00027952	235.661575

Precuneus L	-12	-52.5	70.5	68	0.00027952	173.906342
Precentral R	43.5	0	52.5	173	0.00027952	166.711716
Frontal Sup Medial R	6	49.5	33	21	0.00027952	151.810898
Frontal Sup L	-22.5	12	64.5	54	0.00027952	151.078842
Temporal Mid R	64.5	-4.5	-19.5	97	0.00027952	113.061844
Postcentral R	63	-4.5	22.5	37	0.00027952	109.613739
Temporal Inf R	48	-10.5	-40.5	89	0.00135592	75.7882996
Temporal Pole Mid R	39	21	-33	15	0.00027952	75.1310196
Temporal Pole Mid R	54	10.5	-30	27	0.00027952	73.113884
Frontal Sup R	24	31.5	51	25	0.00027952	47.710804
Parietal Sup R	36	-52.5	57	16	0.00157092	41.7577629
Frontal Mid R	42	27	42	18	0.00070686	37.4673424
Parietal Inf L	-46.5	-34.5	45	46	0.00242265	37.1054192
SupraMarginal R	42	-36	46.5	20	0.00242265	35.1228371

Supplementary Table 10. MSE on minimum-maximum-normalized voxel intensity values by scanner type for FTLD. P_{FDR}<0.05, TFCE-corrected, covariates: age, sex, TIV, and recording site. Regions are presented on MNI space using the AAL atlas.

**Regions**	**T value**	P_{FDR}
Left temporal pole sup - left cerebellum 10	3.33	0.043
Right supp motor area - left precentral	-3.78	0.021
Left supp motor area - left precentral	-3.57	0.039
Left supp motor area - left cerebellum 8	-3.43	0.039
Left temporal pole sup - left cuneus	-3.39	0.043

Left cuneus - left temporal pole sup	-3.39	0.017
Left cuneus - left cerebellum 4 5	-2.95	0.042
Left cuneus - vermis 7	-4.15	0.005
Vermis 1 2 - left calcarine	-4.1	0.006
Left cuneus - right cerebellum 7b	-3.85	0.008
Left cuneus - left cerebellum 6	-3.44	0.017
Left cuneus - left cerebellum crus1	-3.38	0.017
Left cuneus - vermis 6	-3.35	0.017
Left cuneus - left cerebellum crus2	-3.09	0.032
Left cuneus - right cerebellum 6	-3.08	0.032
Vermis 1 2 - right calcarine	-3.44	0.034
Vermis 1 2 - lef lingual	-3.34	0.034
Left temporal pole sup - left paracentral	-3.24	0.043
Left temporal pole sup - right cuneus	-3.19	0.043

Supplementary Table 11. MSE on ROI-to-ROI functional connectivity for AD using the AAL atlas. $P_{FDR}<0.05$, covariates: age, sex, and recording site.

**Regions**	**T value**	$\boldsymbol{P_{FDR}}$
Right precentral - left hippocampus	4.03	0.004
Left precentral - left hippocampus	3.78	0.022
Right supp motor area - left hippocampus	3.2	0.025
Right frontal mid - left hippocampus	3.71	0.027
Right frontal sup - left hippocampus	3.3	0.042
Left supp motor area - left hippocampus	3.05	0.042

Left frontal sup med - left parahippocampal	4.16	0.001
Right frontal sup med - left parahippocampal	3.63	0.019
Right frontal sup orb - left parahippocampal	3.57	0.025
Right frontal sup - left parahippocampal	3.53	0.029
Right supp motor area - left parahippocampal	3.07	0.029
Vermis 3 - left parahippocampal	-2.86	0.047
Left hippocampus - left supp motor area	3.05	0.027
Right amygdala - left supp motor area	3.35	0.027
Right frontal mid - left supp motor area	-3.42	0.027
Left frontal sup med - left supp motor area	-2.96	0.048
Right amygdala - right supp motor area	3.76	0.010
Left frontal sup med - right supp motor area	-3.3	0.021
Left hippocampus - right supp motor area	3.2	0.024
Right frontal mid - right supp motor area	-3.54	0.027
Left amygdala - right supp motor area	3.35	0.027
Right hippocampus - right supp motor area	3.26	0.029
Left parahippocampal - right supp motor area	3.07	0.030
Left hippocampus - right precentral	4.03	0.008
Right hippocampus - right precentral	3.99	0.010
Left rectus - right precentral	-3.23	0.032
Right amygdala - right precentral	3.06	0.032
Right frontal mid - right precentral	-3.18	0.048
Right supp motor area - right amygdala	3.76	0.024
Right precentral - right amygdala	3.06	0.028

Left supp motor area - right amygdala	3.35	0.036
Left precentral - right amygdala	3.21	0.043
Left frontal sup med - right parahippocampal	4.66	0.001
Right frontal sup - right parahippocampal	3.6	0.029
Vermis 3 - right parahippocampal	-2.92	0.047
Right precentral - right hippocampus	3.99	0.004
Right supp motor area - right hippocampus	3.26	0.024
Left precentral - right hippocampus	3.53	0.028
Right supp motor area - left amygdala	3.35	0.024
Left precentral - left amygdala	3.09	0.047
Left hippocampus - left amygdala	-2.7	0.048
Right parahippocampal - left frontal sup med	4.66	0.001
Left rectus - left frontal sup med	4.51	0.001
Left parahippocampal - left frontal sup med	4.16	0.005
Right supp motor area - left frontal sup med	-3.3	0.024
Left supp motor area - left frontal sup med	-2.96	0.042
Left hippocampus - left temporal pole sup	-2.78	0.048
Left parahippocampal - right frontal sup orb	3.57	0.011
Left frontal sup med - right rectus	2.99	0.048
Left hippocampus - right rectus	2.69	0.048
Right parahippocampal - vermis 3	-2.92	0.045
Left parahippocampal - vermis 3	-2.86	0.047
Left parahippocampal - left temporal inf	-3.18	0.027
Left frontal sup med - left rectus	4.51	0.001

Right frontal sup med - left rectus	4.09	0.007
Right precentral - left rectus	-3.23	0.027
Left rectus - right frontal sup med	4.08	0.003
Left parahippocampal - right frontal sup med	3.63	0.011
Right supp motor area - right frontal sup med	-3.05	0.029
Left supp motor area - right frontal sup med	-2.94	0.042
Right parahippocampal - right frontal sup med	2.91	0.045
Left hippocampus - right frontal sup med	2.77	0.048
Left hippocampus - left precentral	3.78	0.010
Right hippocampus - left precentral	3.53	0.026
Right amygdala - left precentral	3.21	0.028
Left amygdala - left precentral	3.09	0.045
Right parahippocampal - left caudate	3.23	0.022
Right precentral - left caudate	-2.93	0.036
Right supp motor area - right temporal pole sup	3.15	0.026
Right parahippocampal - right frontal sup	3.6	0.011
Left parahippocampal - right frontal sup	3.53	0.011
Left hippocampus - right frontal sup	3.3	0.024
Left hippocampus - right frontal mid	3.71	0.010
Right supp motor area - right frontal mid	-3.54	0.024
Right precentral - right frontal mid	-3.18	0.027
Left supp motor area - right frontal mid	-3.42	0.036
Left temporal pole sup - left cingulum mid	4.77	0.000
Left frontal sup med - left frontal med orb	4.28	0.001

Right amygdala - left cingulum mid	4.21	0.004
Right precentral - left frontal sup orb	-3.94	0.004
Right parahippocampal - left cingulum ant	4	0.005
Left parahippocampal - left cingulum ant	3.88	0.008
Vermis 9 - left cerebellum 6	-3.86	0.009
Vermis 9 - right supramarginal	3.82	0.009
Right amygdala - left parietal sup	3.7	0.010
Right precentral - vermis 4 5	3.62	0.010
Right parahippocampal - right occipital inf	-3.66	0.011
Left frontal sup med - right frontal med orb	3.49	0.013
Left rectus - right postcentral	-3.62	0.013
Right parahippocampal - left cingulum mid	3.46	0.015
Left supp motor area - left rolandic oper	3.88	0.015
Left amygdala - left postcentral	3.8	0.016
Left amygdala - left supramarginal	3.68	0.016
Left rectus - left frontal sup	3.48	0.017
Right frontal sup orb - left cerebellum 3	3.83	0.018
Left hippocampus - right postcentral	3.44	0.019
Vermis 3 - left frontal sup orb	3.74	0.022
Vermis 3 - left occipital mid	-3.6	0.022
Right parahippocampal - right fusiform	-3.24	0.022
Right parahippocampal - right temporal pol mid	-3.2	0.022
Right temporal pole sup - left cingulum mid	3.79	0.022
Vermis 9 - right putamen	3.47	0.023

Right supp motor area - left rolandic oper	3.34	0.024
Left hippocampus - right cingulum mid	3.26	0.024
Left hippocampus - left postcentral	3.17	0.024
Right precentral - right frontal mid orb	-3.31	0.024
Right frontal sup orb - right postcentral	-3.45	0.025
Right hippocampus - left postcentral	3.44	0.026
Right hippocampus - right postcentral	3.35	0.027
Left hippocampus - right precuneus	3.11	0.027
Left hippocampus - left cerebellum crus1	-3.05	0.027
Left amygdala - left parietal inf	3.35	0.027
Left parahippocampal - right frontal inf orb	3.21	0.027
Right precentral - right rolandic oper	3.1	0.028
Right precentral - right insula	3.1	0.028
Right amygdala - left postcentral	3.25	0.028
Left precentral - right lingual	3.4	0.030
Right frontal inf oper - right fusiform	3.48	0.030
Right frontal inf oper - left cerebellum 10	3.44	0.030
Right frontal inf oper - left fusiform	3.4	0.030
Left parahippocampal - right cingulum ant	3.09	0.030
Right amygdala - left parietal inf	3.08	0.032
Right amygdala - left frontal sup	3.05	0.032
Left parahippocampal - left temporal pol mid	-2.99	0.035
Vermis 9 - left cerebellum 7b	-3.27	0.036
Right precentral - left frontal med orb	-2.88	0.036

Right precentral - left rolandic oper	2.86	0.036
Right precentral - right lingual	2.84	0.036
Right precentral - right caudate	-2.83	0.036
Right precentral - right cingulum ant	-2.83	0.036
Right frontal inf oper - left temporal pol mid	3.25	0.037
Vermis 9 - vermis 1 2	-3.13	0.039
Vermis 9 - left temporal pol mid	-3.12	0.039
Vermis 9 - left cerebellum 10	-3.05	0.041
Left supp motor area - left frontal sup	-3.12	0.042
Left supp motor area - right lingual	3.02	0.042
Left supp motor area - right rolandic oper	3.01	0.042
Left supp motor area - right putamen	2.95	0.042
Left hippocampus - right cerebellum crus1	-2.85	0.045
Left amygdala - left pallidum	3.07	0.045
Right parahippocampal - left temporal pol mid	-2.88	0.046
Left precentral - left lingual	3.06	0.047
Left parahippocampal - right cingulum mid	2.83	0.047
Vermis 3 - right caudate	3.22	0.047
Vermis 3 - left frontal mid orb	3.12	0.047
Vermis 3 - right occipital mid	-3.06	0.047
Vermis 3 - left cerebellum crus1	-2.96	0.047
Vermis 3 - left fusiform	-2.95	0.047
Vermis 3 - vermis 10	-2.9	0.047
Vermis 3 - right occipital sup	-2.87	0.047

Left hippocampus - right paracentral lob	2.72	0.048
Left hippocampus - left paracentral lob	2.72	0.048

Supplementary Table 12. MSE on ROI-to-ROI functional connectivity for FTLD using the AAL atlas. $P_{FDR}<0.05$, covariates: age, sex, and recording site.

Dimension	CFI	TLI	RMSEA	SRMR
Education	0.995	0.965	0.053	0.013
Food insecurity	0.988	0.907	0.107	0.017
Financial status	0.985	0.975	0.035	0.026
Assets	0.943	0.883	0.081	0.044
Access to healthcare	0.993	0.985	0.023	0.018
Childhood labor	0.998	0.974	0.073	0.017
Subjective SES	0.99	0.99	0.001	0.001
Childhood experiences	0.883	0.805	0.086	0.087
Traumatic events	0.933	0.918	0.03	0.032
Relations	0.998	0.995	0.02	0.019

Supplementary Table 13. Fit indices of the validation analysis of dimensions. CFI = Comparative Fit Index; TLI = Tucker-Lewis Index; RMSEA = Root Mean Square Error of Approximation; SRMR = Standardized Root Mean Square Residual.

		HC	AD	FTLD
Sample size		442	310	123
Country	Argentina	38	44	2
	Brazil	69	13	10

	Chile	53	58	26
	Colombia	25	133	45
	Mexico	29	19	5
	Peru	228	43	35
Age		60.69 (11.45)	70.62 (7.73)	67.23 (7.23)
Sex (F:M)		318:124	196:114	63:60
Cognition (MMSE)		26.34 (3.77)	21.29 (4.71)	21.84 (5.40)
Functional ability (Pfeffer)		0.21 (1.50)	10.70 (8.32)	12.72 (8.98)
Neuropsychiatric symptoms (NPI-Q)		1.18 (2.27)	5.72 (5.25)	10.48 (7.04)

Supplementary Table 14. Demographic, cognitive, functional, and neuropsychiatric characterization of the MRI subsample.

		HC	AD	FTLD
Sample size		183	232	85
Country	Argentina	27	23	1
	Brazil	65	11	10
	Chile	47	56	24
	Colombia	24	133	45
	Mexico	20	9	5
	Peru	0	0	0
Age		57.37 (14.14)	70.78 (7.44)	67.27 (7.01)

Sex (F:M)	123:60	141:91	45:40
Cognition (MMSE)	28.61 (1.53)	21.20 (4.56)	22.07 (5.30)
Functional ability (Pfeffer)	0.30 (2.18)	11.48 (8.45)	11.93 (8.65)
Neuropsychiatric symptoms (NPI-Q)	1.56 (2.92)	5.93 (5.21)	10.99 (6.83)

Supplementary Table 15. Demographic, cognitive, functional, and neuropsychiatric characterization of the resting-state fMRI subsample.

Association	β	P value	Lower 5% C	Upper 95% CI
All subjects model (CFI = 0.945, TLI = 0.905, RMSEA = 0.069, SRMR = 0.045)				
MSE → Cognition	0.239	1×10^{-308}	0.197	0.282
MSE → Functional ability	0.056	0.012	0.012	0.101
MSE → Neuropsychiatric symptoms	0.065	0.004	0.021	0.109
HC model (CFI = 0.874, TLI = 0.781, RMSEA = 0.095, SRMR = 0.081)				
MSE → Cognition	0.564	1×10^{-11}	0.518	0.61
MSE → Functional ability	0.029	0.316	-0.028	0.087
MSE → Neuropsychiatric symptoms	-0.045	0.121	-0.103	0.012
AD model (CFI = 0.964, TLI = 0.937, RMSEA = 0.051, SRMR = 0.041)				
MSE → Cognition	0.216	1×10^{-7}	0.149	0.282
MSE → Functional ability	0.069	0.048	0.001	0.137
MSE → Neuropsychiatric symptoms	0.069	0.047	0.001	0.137
FTLD model (CFI = 0.981, TLI = 0.968, RMSEA = 0.038, SRMR = 0.043)				
MSE → Cognition	0.149	0.025	0.019	0.279
MSE → Functional ability	0.152	0.022	0.021	0.282

MSE → Neuropsychiatric symptoms	0.153	0.021	0.023	0.283

Supplementary Table 16. Models results without applying imputation strategies for missing values. No adjustments were made for multiple comparisons. The SEM were implemented as two-sided models by default.

Location	Scanner Model Tesla	Seq name	TR (ms)	TE (ms)	Flip angle (°)	Matrix dim	Voxel size (mm)
Argentina site	1.5T GE Signa HDxt	T1 3D FFE	0.008824	0.003548	13	256x256x156	0.95x0.95x1
Brazil site 1	3T Siemens Verio	SAG T1 3D	1.8	0.00244	9	224x512x512	0.5x0.5x0.5
Brazil site 2	3T Philips Achieva	T1 3D FFE	0.0064768	0.002949	9	192x256x256	1x1x1
Chile site 1	3T Philips Ingenia	T1 3D FFE	0.0077732	0.003553	8	355x576x576	0.5x0.5x0.5
Chile site 2	3T Siemens MAGNETOM Lumina	MP RAGE	1.8	0.00213	8	192x256x256	1x1x1
Chile site 3	3T Philips Ingenia	T1 3D FFE	0.0078661	0.003601	8	355x576x576	0.5x0.5x0.5
Chile site 4	3T Siemens MAGNETOM Lumina	T1 3D FFE	1.8	0.00213	8	192x256x256	1x1x1

Colombia site 1	3T Siemens Skyra	MP RAGE	2.3	0.00225	8	192x256x256	0.95x0.95x0.95
Colombia site 2	3T Philips Ingenia Elition X	TFE	0.0080664	0.003694	8	200x256x256	1x1x1
Colombia site 3	3T Philips Achieva	SAG T1 3D	0.0076217	0.003747	8	180x256x256	1x1x1
Mexico site	3T Siemens Biograph mMR	MP RAGE	2.3	0.00289	9	160x256x256	1x1x1
Peru site 1	3T Siemens Skyra	MP RAGE	2.3	0.00227	8	176x256x256	1x.977x.977
Peru site 2	3T Siemens Spectra	MP RAGE	1.9	0.00242	9	160x256x256	1x.977x.977

Supplementary Table 17. Structural MRI acquisition parameters per site.

Location	Scanner Model Tesla	TR (ms)	TE (ms)	Voxel size (mm)	N° vol	Matrix dimension	Flip angle (º)	N° slices
Argentina site	1.5T GE Signa HDxt	2.5	0.03	3.8x3.8x5	120	64x33	50	33
Brazil site 1	3T Siemens Verio	3	0.03	3.4x3.4x5	80	64x30	90	36

Brazil site 2	3T Philips Achieva	2.2	0.028001	3.3x3.3x3.3	275	64x44	80	44
Chile site 1	3T Philips Ingenia	4.9715	0.030001	2.5x2.5x2.8	121	96x45	82	45
Chile site 2	3T Siemens MAGNETOM Lumina	2.5	0.03	2.5x2.5x2.5	235	94x66	90	66
Chile site 3	3T Philips Ingenia	4.97133	0.03	2.5x2.5x2.8	121	96x45	82	45
Chile site 4	3T Siemens MAGNETOM Lumina	2.5	0.03	2.5x2.5x2.5	235	94x66	90	66
Colombia site 1	3T Siemens Skyra	3	0.03	3x3x3	200	70x45	80	39
Colombia site 2	3T Philips Ingenia Elition X	2.5	0.03	3x3x3	240	80x60	90	60
Colombia site 3	3T Philips Achieva	3	0.03	3x3x3	197	64x48	90	50
Mexico site	3T Siemens Biograph mMR	3.5	0.032	2.2x2.2x2.7	120	94x66	45	66

Supplementary Table 18. Resting-state fMRI acquisition parameters per site.

References

1 Shipley, B. *Cause and Correlation in Biology: A User's Guide to Path Analysis, Structural Equations and Causal Inference with R*. 2 edn, (Cambridge University Press, 2016).

2 Byrne, B. M. *Structural Equation Modeling with EQS and EQS-Windows: Basic Concepts, Applications, and Programming*. (Sage Publications, Inc., 1994).

3 Kline, R. B. *Principles and practice of structural equation modeling, 4th ed*. (Guilford Press, 2016).

4 Shi, D., Lee, T. & Maydeu-Olivares, A. Understanding the Model Size Effect on SEM Fit Indices. *Educational and psychological measurement* **79**, 310-334, doi:10.1177/0013164418783530 (2019).

5 Aknin, L. B. *et al.* Policy stringency and mental health during the COVID-19 pandemic: a longitudinal analysis of data from 15 countries. *The Lancet Public Health* **7**, e417-e426, doi:10.1016/S2468-2667(22)00060-3 (2022).

6 Sekher, T. V., Pai, M. & Muhammad, T. Subjective social status and socio-demographic correlates of perceived discrimination among older adults in India. *BMC geriatrics* **24**, 617, doi:10.1186/s12877-024-05114-x (2024).

7 Nylund, K. L., Asparouhov, T. & Muthén, B. O. Deciding on the Number of Classes in Latent Class Analysis and Growth Mixture Modeling: A Monte Carlo Simulation Study. *Structural Equation Modeling: A Multidisciplinary Journal* **14**, 535-569, doi:10.1080/10705510701575396 (2007).

8 Nieto-Castanon, A. *Handbook of functional connectivity Magnetic Resonance Imaging methods in CONN*. (2020).

9 Tzourio-Mazoyer, N. *et al.* Automated anatomical labeling of activations in SPM using a macroscopic anatomical parcellation of the MNI MRI single-subject brain. *Neuroimage* **15**, 273-289, doi:10.1006/nimg.2001.0978 (2002).

10 Fan, L. *et al.* The Human Brainnetome Atlas: A New Brain Atlas Based on Connectional Architecture. *Cerebral Cortex* **26**, 3508-3526, doi:10.1093/cercor/bhw157 (2016).
