# Supplementary material for: Social exposome and brain health outcomes of dementia across Latin America
Source: Nat Commun. 2025 Sep 11;16:8196. doi: 10.1038/s41467-025-63277-6 (PMC12426228; doi:10.1038/s41467-025-63277-6)
Supplement: Supplementary file 2 — Reporting Summary [file 41467_2025_63277_MOESM2_ESM.pdf]

nature portfolio

Corresponding author(s): Agustin Ibanez

Last updated by author(s): Jul 3, 2025

Reporting Summary

Nature Portfolio wishes to improve the reproducibility of the work that we publish. This form provides structure for consistency and transparency in reporting. For further information on Nature Portfolio policies, see our Editorial Policies and the Editorial Policy Checklist.

Statistics

For all statistical analyses, confirm that the following items are present in the figure legend, table legend, main text, or Methods section.

n/a	Confirmed	
☐	☒	The exact sample size (*n*) for each experimental group/condition, given as a discrete number and unit of measurement
☐	☒	A statement on whether measurements were taken from distinct samples or whether the same sample was measured repeatedly
☐	☒	The statistical test(s) used AND whether they are one- or two-sided<br>*Only common tests should be described solely by name; describe more complex techniques in the Methods section.*
☐	☒	A description of all covariates tested
☐	☒	A description of any assumptions or corrections, such as tests of normality and adjustment for multiple comparisons
☐	☒	A full description of the statistical parameters including central tendency (e.g. means) or other basic estimates (e.g. regression coefficient) AND variation (e.g. standard deviation) or associated estimates of uncertainty (e.g. confidence intervals)
☐	☒	For null hypothesis testing, the test statistic (e.g. *F*, *t*, *r*) with confidence intervals, effect sizes, degrees of freedom and *P* value noted<br>*Give P values as exact values whenever suitable.*
☒	☐	For Bayesian analysis, information on the choice of priors and Markov chain Monte Carlo settings
☒	☐	For hierarchical and complex designs, identification of the appropriate level for tests and full reporting of outcomes
☐	☒	Estimates of effect sizes (e.g. Cohen's *d*, Pearson's *r*), indicating how they were calculated

Our web collection on statistics for biologists contains articles on many of the points above.

Software and code

Policy information about availability of computer code

Data collection	no software was used.
Data analysis	CAT12 (https://neuro-jena.github.io/cat/), SPM12 (https://www.fil.ion.ucl.ac.uk/spm/software/spm12/), fmriPrep (v22.0.2) and CONN (v 22.a) were used in MATLAB 2022a to process imaging data. Nilearn library (v0.10.0) in Python (v3.8, Python Software Foundation) was used to extract whole-brain gray matter volume. Structural equation modelling, Lasso, and meta-regression analyses were run with in R (v 2024.04.2). MRIcroGL (v1.2.20220720), Brain Net Viewer (v 1.7), and CONN (v 22.a) was used to plot brain imaging results. Scripts used to perform analyses are available at OSF repository https://osf.io/78ng6/.

For manuscripts utilizing custom algorithms or software that are central to the research but not yet described in published literature, software must be made available to editors and reviewers. We strongly encourage code deposition in a community repository (e.g. GitHub). See the Nature Portfolio guidelines for submitting code & software for further information.

Data

Policy information about availability of data

All manuscripts must include a data availability statement. This statement should provide the following information, where applicable:

- Accession codes, unique identifiers, or web links for publicly available datasets
- A description of any restrictions on data availability
- For clinical datasets or third party data, please ensure that the statement adheres to our policy

The preprocessed behavioral, MRI, and fMRI data generated in this study have been deposited in the OSF database under accession code https://osf.io/78ng6/. The

raw data are available under restricted access for ethical and regulatory constraints. Access can be obtained after IRB approval of the formal data-sharing agreement in a process that can last up to 12 weeks. For questions related to the data request and usage, contact Agustín Ibañez at agustin.ibanez@gbhi.org.

Research involving human participants, their data, or biological material

Policy information about studies with human participants or human data. See also policy information about sex, gender (identity/presentation), and sexual orientation and race, ethnicity and racism.

Reporting on sex and gender	Findings include both sexes. Sex was used as covariate in our analyses. The study included 2,211 participants with a mean age of 64.63 years (SD = 11.26), of whom 67.03% were women. 3D T1-weighted images were collected for 875 individuals with a mean age of 65.13 years (SD = 10.77), of whom 65.94% were women. Resting-state sequences were collected for 500 individuals with a mean age of 65.27 years (SD = 12.02), of whom 61.80% were women. Sex information was determined by self-report.
Reporting on race, ethnicity, or other socially relevant groupings	Participants were recruited from the Multi-Partner Consortium to Expand Dementia Research in Latin America (ReDLat)111, with recruitment conducted across six LA countries: Argentina (n = 112, HC:AD/FTLD = 52:60), Brazil (n = 172, HC:AD/FTLD = 116:56), Chile (n = 200, HC:AD/FTLD = 78:122), Colombia (n = 730, HC:AD/FTLD = 250:480), Mexico (n = 356, HC:AD/FTLD = 227:129), Peru (n = 641, HC:AD/FTLD = 452:189)
Population characteristics	The sample was comprised of individuals with probable AD (n = 781), FTLD (n = 255), and HC (n = 1,175). Exclusion criteria included participants with conditions other than AD or FTLD or those with impairments preventing task completion. Diagnoses were determined by consensus among expert healthcare providers at each site, based on cognitive and neurological exams, clinical interviews, and MRI. The diagnoses are based on the clinical criteria established by the National Institute of Neurological and Communicative Disorders and Stroke and the Alzheimer's Disease and Related Disorders Association (NINCDS-ADRDA) for AD, as well as on the clinical criteria specified for FTLD. Healthy Controls had preserved cognition and no history of neurological or psychiatric conditions.
Recruitment	Participants were recruited from extensive networks including (a) clinical networks, involving memory clinics, neurology departments, and affiliated hospitals; (b) academic collaborations, leveraging partnerships with universities and research institutions; (c) community outreach programs, engaging with local communities through informational sessions, and culturally tailored materials to encourage participation from rural and urban populations with diverse socioeconomic backgrounds; and (d) public health initiatives and local organizations, integrating recruitment efforts with public health campaigns and community groups to raise awareness and facilitate participation. These efforts allowed us to include individuals from rural and urban settings, focusing on underrepresented groups, as demonstrated previously with our ReDLat cohort marked by socioeconomic inequality and educational disparities. Strategies to improve access and recruitment for these groups involve field screenings, community engagement efforts, and the use of mobile units. No compensation was provided to participants.
Ethics oversight	The institutional review boards of each recruitment site and the Executive Committee of the ReDLat consortium approved this study. All participants signed informed consent in accordance with the Declaration of Helsinki.

Note that full information on the approval of the study protocol must also be provided in the manuscript.

Field-specific reporting

Please select the one below that is the best fit for your research. If you are not sure, read the appropriate sections before making your selection.

[x] Life sciences   [ ] Behavioural & social sciences   [ ] Ecological, evolutionary & environmental sciences

For a reference copy of the document with all sections, see nature.com/documents/nr-reporting-summary-flat.pdf

Life sciences study design

All studies must disclose on these points even when the disclosure is negative.

Sample size	We aimed to reach the N:q ratio of 10:1 to achieve statistical precision and power for the SEM analysis. Even the smallest sample size (FTLD) met the N ratio of 10:1 for statistical accuracy and power, with n = 225 participants exceeding the number of estimated parameters (q = 13).
Data exclusions	Data was excluded when the complete multidimensional social exposome questionnaire was missing.
Replication	Reproducibility of experimental findings are ensured by data and code availability documented at the manuscript. Also, results were replicated through different statistical methods rigorously applied using standard software packages.
Randomization	This is an observational, cross-sectional, quantitative study designed to examine the impact of multidimensional social exposome on brain health outcomes in aging and dementia across Latin American populations. Therefore, samples allocation was not random. Randomization was not applicable. They were grouped by by diagnosis (healthy controls, frontotemporal lobe degeneration, Alzheimer's disease). Covariates in analyses included age, sex, disease severity, age at diagnosis, years after diagnosis, FTLD subtype (when applicable), total intracraneal volume and scanner type. Potential self-selection bias may be introduced due to voluntary participation, which may limit generalizability to more socially advantaged individuals. This could lead to underestimation of associations between social disadvantage and brain health outcomes.
Blinding	This study used pre-existing data. Neuroimages and multidimensional social exposome data were obtained from the Multi-Partner Consortium

to Expand Dementia Research in Latin America [ReDLat, https://red-lat.com/]. Blinding was not necessary for this study because it used pre-existing data from neuroimaging repositories and social exposome datasets, with no direct participant interaction or potential for intervention bias.

Reporting for specific materials, systems and methods

We require information from authors about some types of materials, experimental systems and methods used in many studies. Here, indicate whether each material, system or method listed is relevant to your study. If you are not sure if a list item applies to your research, read the appropriate section before selecting a response.

Materials & experimental systems

n/a	Involved in the study
[x]	[ ] Antibodies
[x]	[ ] Eukaryotic cell lines
[x]	[ ] Palaeontology and archaeology
[x]	[ ] Animals and other organisms
[x]	[ ] Clinical data
[x]	[ ] Dual use research of concern
[x]	[ ] Plants

Methods

n/a	Involved in the study
[x]	[ ] ChIP-seq
[x]	[ ] Flow cytometry
[ ]	[x] MRI-based neuroimaging

Plants

Seed stocks	n/a
Novel plant genotypes	n/a
Authentication	n/a

Magnetic resonance imaging

Experimental design

Design type	structural MRI and resting-state fMRI
Design specifications	one MRI/fMRI session per subject
Behavioral performance measures	n/a; active fMRI tasks were not used in this study.

Acquisition

Imaging type(s)	Structural 3D-T1 weighted and resting-state sequences
Field strength	1.5 and 3 tesla
Sequence & imaging parameters	Multiple scanners were involved. Sequence & imaging parameters were reported in Supplementary information.
Area of acquisition	whole-brain scans

Diffusion MRI [ ] Used [x] Not used

Preprocessing

Preprocessing software	MRI preprocessing and analysis were employed using VBM with the Computational Anatomy (CAT12) toolbox and in Statistical Parametric Mapping software (SPM 12; Wellcome Centre for Human Neuroimaging; www.fil.ion.ucl.ac.uk/spm/software/spm12/) in Matlab R2021a. The standard pipeline included bias-field correction, noise reduction, skull stripping, segmentation, and normalization to the Montreal Neurological Institute (MNI) space at a 1.5 mm isotropic resolution. CAT12 also performed intra-subject harmonization by normalizing data to the mean global intensity for each subject, followed by smoothing gray matter segmentations with a 6 x 6 x 6 mm Gaussian kernel. Homogeneity and orthogonality of the images were verified. Scanner effects were controlled through two approaches: by including scanner type as a covariate in the VBM

	regression models and by standardizing between the minimum and maximum intensity values of each voxel for all subjects evaluated by each scanner type.<br><br>fMRI image preprocessing was employed using the fmriprep (version 22.0.2) standard pipeline, encompassing head motion artifacts, slice timing, susceptibility distortion correction, co-registration to the anatomical image, and normalization to standard space with additional steps in the CONN22.a toolbox. This involved smoothing with a 6 x 6 x 6 mm Gaussian kernel and denoising through linear regression with nine nuisance regressors applied in a single step: six motion parameters (translation and rotation), white matter, cerebrospinal fluid signals, and scrubbing regressors for high-motion time points, and applying a band-pass filter (0.008-0.09 Hz. A motion correction technique was applied by rigidly aligning fMRI volumes to T1-weighted images, ensuring that the impact of motion artifacts was minimized. Motion scrubbing was then applied using framewise displacement > 0.2 mm and temporal derivative variance across apace> 5%, which are stricter than conventional thresholds, to flag and remove high-motion frames. The mean proportion of artifact-free to rejected frames was 0.915 (SD = 0.126), with values ranging from 30% to 100%. This was implemented using the artifact detection tools within the CONN toolbox, using a conservative setting (FD = 0.2 mm, global signal Z = 5) to remove motion artefacts while preserving the biological signal. Pearson correlation coefficients were computed between the average BOLD time series of each pair of regions of interest (ROIs) from the Brainnetome atlas, a structural and functional connectivity-based parcellation atlas that capture both cortical and subcortical regions, better suited to functional connectivity analysis. AAL atlas cerebellar regions were added, generating a total of 272 x 272 ROIs correlation matrix for each participant. These correlation matrices were Fisher z-transformed to normalize the distribution of the correlation coefficients. Scanner variability was controlled by including scanner type as a covariate in the ROI-to-ROI connectivity regression models.
Normalization	MRI normalization was employed via standard pipeline, including bias-field correction, noise reduction, skull stripping, segmentation, and normalization to the Montreal Neurological Institute (MNI) space at a 1.5 mm isotropic resolution. CAT12 also performed intra-subject harmonization by normalizing data to the mean global intensity for each subject, followed by smoothing gray matter segmentations with a 6 x 6 x 6 mm Gaussian kernel. Homogeneity and orthogonality of the images were verified.<br><br>FMRI image normalization was employed via standard pipeline, encompassing head motion artifacts, slice timing and susceptibility distortion correction, co-registration to the anatomical image, and normalization to standard space with additional steps in the CONN22.a toolbox. This involved smoothing with a 6 x 6 x 6 mm Gaussian kernel, denoising through linear regression to remove confounding factors (white matter, cerebrospinal fluid, head motion parameters, and scrubbing), and applying a band-pass filter (0.008-0.09 Hz).
Normalization template	Standard MNI152.
Noise and artifact removal	MRI: we employed bias-field correction, noise reduction, skull stripping, segmentation, and normalization to the Montreal Neurological Institute (MNI) space at a 1.5 mm isotropic resolution. CAT12 also performed intra-subject harmonization by normalizing data to the mean global intensity for each subject, followed by smoothing gray matter segmentations with a 6 x 6 x 6 mm Gaussian kernel. Homogeneity and orthogonality of the images were verified.<br><br>fMRI: we employed head motion artifacts, slice timing and susceptibility distortion correction, co-registration to the anatomical image, and normalization to standard space with additional steps in the CONN22.a toolbox. This involved smoothing with a 6 x 6 x 6 mm Gaussian kernel and denoising through linear regression with nine nuisance regressors applied in a single step: six motion parameters (translation and rotation), white matter, cerebrospinal fluid signals, and scrubbing regressors for high-motion time points, and applying a band-pass filter (0.008-0.09 Hz. A motion correction technique was applied by rigidly aligning fMRI volumes to T1-weighted images, ensuring that the impact of motion artifacts was minimized. Motion scrubbing was then applied using framewise displacement > 0.2 mm and temporal derivative variance across apace> 5%, which are stricter than conventional thresholds, to flag and remove high-motion frames. The mean proportion of artifact-free to rejected frames was 0.915 (SD = 0.126), with values ranging from 30% to 100%. This was implemented using the artifact detection tools within the CONN toolbox, using a conservative setting (FD = 0.2 mm, global signal Z = 5) to remove motion artefacts while preserving the biological signal
Volume censoring	Motion scrubbing was then applied using framewise displacement > 0.2 mm and temporal derivative variance across apace> 5%, which are stricter than conventional thresholds, to flag and remove high-motion frames

Statistical modeling & inference

Model type and settings	The model's structure was based on ten composite variables derived from the MSE questionnaire, which included education, nutrition, financial status, assets, access to healthcare (randomly set as the fixed indicator to identify the values for the latent factor) childhood labor, subjective SES, childhood experiences, traumatic events, and relationships. These variables served as indicators of a single MSE latent variable, which was used to predict cognition, functional ability, and neuropsychiatric symptoms.<br>Lasso regression was employed to test the contributions of individual predictors across outcomes and then compare the effects with the global MSE score via meta-regression. We opted for this approach due to its capacity to deal with several variables that tend to be collinear, resulting in simpler and more straightforward models. The predictors included the composite factors for education, nutrition, financial status, assets, healthcare access, childhood labor, subjective SES, childhood experiences, traumatic events, and relationships as predictors. Lasso model was tuned using cross-validation to determine the optimal regularization parameter that minimized the prediction error.<br>Bootstrap resampling (n = 400) was applied to estimate the standardized beta coefficients for the paths from the MSE factor to each outcome, capturing the effect sizes and their variability across the bootstrap samples.
Effect(s) tested	SEM: childhood labor, subjective SES, childhood experiences, traumatic events, and relationships served as indicators of a single MSE latent variable, which was used to predict cognition, functional ability, and neuropsychiatric symptoms.<br>Lasso: The predictors included the composite factors for education, nutrition, financial status, assets, healthcare access,

childhood labor, subjective SES, childhood experiences, traumatic events, and relationships as predictors of cognition, functional ability, and neuropsychiatric symptoms.
MRI: We conducted regression analyses using parametric tests to examine the associations between MSE and brain volume.
fMRI: We conducted regression analyses via parametric tests with the MSE score as predictor of the whole-brain ROI-to-ROI connectivity

Specify type of analysis: [x] Whole brain [ ] ROI-based [ ] Both

Statistic type for inference (See Eklund et al. 2016)

Threshold-free cluster-wise inference method, applied via the TFCE toolbox (http://www.neuro.uni-jena.de/tfce)

Correction

false discovery rate (FDR) correction of $P < 0.05$

Models & analysis

n/a	Involved in the study
[ ]	[x] Functional and/or effective connectivity
[x]	[ ] Graph analysis
[x]	[ ] Multivariate modeling or predictive analysis

Functional and/or effective connectivity

Pearson correlation coefficients were computed between the average BOLD time series of each pair of regions of interest (ROIs) from the Brainnetome atlas, a structural and functional connectivity-based parcellation atlas that capture both cortical and subcortical regions, better suited to functional connectivity analysis. AAL atlas cerebellar regions were added, generating a total of 272 x 272 ROIs correlation matrix for each participant. These correlation matrices were Fisher z-transformed to normalize the distribution of the correlation coefficients
